# Establishing a Dedicated Fund to Improve Patient Access to Cancer Medicines: Key Considerations and Policy Implications for Thailand: Comment on "Scoping Review of International Experience of a Dedicated Fund to Support Patient Access to Cancer Drugs: Policy Implications for Thailand"

**DOI:** 10.34172/ijhpm.9153

**Published:** 2025-12-03

**Authors:** Jong Hyuk Lee

**Affiliations:** College of Pharmacy, Chung-Ang University, Seoul, Republic of Korea.

**Keywords:** Cancer Drugs Fund, Access to Medicines, Innovative Cancer Drugs, Health Policy, Thailand

## Abstract

Thai policy-makers should carefully design a well-structured, managed-access cancer drug fund (CDF) that aligns with the country’s socioeconomic system and complements existing institutional and policy frameworks. Developing this framework requires a clear and evidence-based definition of innovative cancer drugs, grounded in measurable clinical benefits such as overall survival (OS) and cost-effectiveness. It also requires careful consideration of alternative policies to address the challenges posed by high-priced cancer drugs. In addition, strategies are needed to incorporate cost-ineffective yet life-saving drugs with no therapeutic alternatives into the healthcare system in a fair and transparent manner. It also requires rigorous evaluation of the effectiveness of the CDF, supported by empirical evidence and underpinned by robust operational and data governance frameworks. Such a framework could serve as a foundation for Thailand’s future efforts to balance innovation, affordability, and equity in cancer care.

 Luksameesate and colleagues’ study, published in 2024 builds on the idea that cancer drug funds (CDFs) can advance Pareto efficiency, given the large population of cancer patients suffering from the lack of affordable treatment options, and decision-makers in Thailand need to draw lessons from other countries operating CDFs, Hong Kong, England, and Italy, as valuable inputs. A CDF is a ring-fenced fund for innovative cancer drugs that have not been cleared yet for clinical uncertainties and/or reimbursement decisions and possibly for medicines for rare diseases, with the aim of either providing provisional financial assistance or early access.

 Regarding several points that require clarification, some revisions would help enhance the clarity and credibility of the study. Since the topic centers on governance and regulatory mechanisms, the paper should rely primarily on grey literature rather than on secondary academic sources that merely cite them. Official government data provide the most up-to-date and authoritative figures; for instance, England’s CDF currently covers 102 cancer drugs, not 95 as stated in the paper.^1^ It should also be clarified that the authors reviewed materials in local languages, since full policy documents are usually published in those languages. Furthermore, all numerical data should specify observation periods and data ranges to ensure transparency. Finally, it would enrich the paper to include a comparison with similar funding mechanisms for rare diseases and high-cost medicines in other countries.

 Second, the paper still lacks a comprehensive description of the overall scope of cancer drug approval and reimbursement processes, as well as related ancillary measures, which would provide a clearer understanding of the Thai government’s position on its state-initiated efforts to improve accessibility. The CDF is only one of the policy tools that countries can employ within their budgetary frameworks. A more holistic understanding would help identify the grey areas that have not yet been addressed by current policy initiatives and clarify how different policy measures interact in practice in Thailand. A one-to-one comparison of the detailed evaluation metrics, such as cutoffs and thresholds, should also be conducted.

 Third, given that “accessibility” encompasses both affordability and timeliness, evaluations of dedicated cancer funds should adopt a two-layered approach. There is a growing body of literature examining the efficiency and value of CDFs, which can be broadly divided into two distinct streams: those focusing on clinical benefits and those emphasizing early access. Aggarwal et al evaluated the CDF in England using six value criteria, including clinical benefit scales, cost-effectiveness thresholds defined by NICE (National Institute for Health and Care Excellence), and assessments based on real-world data (RWD).^2^ They argued that, as of January 2015, only a small fraction of CDF-approved indications—38% (18 out of 47)—offered clinical benefits such as overall survival (OS), while more than half—55% (29 out of 47)—failed to pass NICE appraisal, suggesting that the CDF did not deliver the expected value to society. Meanwhile, Sabry-Grant et al praised the CDF for improving early access for patients in need, based on data from May 2018.^3^

 Last, certain expressions in the paper may cause confusion in their interpretation. When the authors stated that CDFs in England provide early access for cancer drugs that have “received a negative recommendation from NICE” or are “unapproved by NICE,” this actually means that such drugs are provisionally recommended by NICE to be commissioned within the CDF framework rather than through the regular reimbursement routes. In other words, CDFs handle cancer drugs that receive provisional positive recommendations from NICE.^1,4^ According to the National Health Service, out of 57 Managed Access Agreements reached between biopharmaceutical companies and the CDF, NICE has reappraised 30 treatments, of which 26 (87%) have been recommended for routine commissioning.^1^

 This commentary adopts a structured approach to these issues. To enhance coherence, the discussion follows four analytical steps: defining innovative cancer drugs, examining alternative access policies, considering the role of cost-effectiveness, and evaluating the effectiveness of CDFs.

## Definition of Innovative Cancer Drugs

 Innovative cancer drugs should be characterized by measurable clinical benefits and supported by robust evidence of added therapeutic value, in line with multicriteria evaluation frameworks such as those used by Agenzia Italiana del Farmaco in Italy.^5^ Recognition through expedited pathways, such as the U.S. Food and Drug Administration (FDA) Breakthrough Therapy designation and the European Medicines Agency (EMA) Priority Medicines program, can also serve as a pragmatic proxy for innovation, as these programs require evidence of substantial improvement over existing therapies for serious conditions.^6^ Furthermore, to ensure conceptual clarity, it is necessary to clearly define what is meant by “innovative status,” “conditional innovative,” and “not innovative.” The technical definition of innovativeness is one of the key attributes of CDFs against which cross-country comparisons are made. Additionally, the Thai policy discussion should begin with a precise understanding of what qualifies as “innovative,” as this definition affects pricing, reimbursement, clinical priority-setting, and eligibility for any dedicated cancer fund.

## Alternative Policy Approaches to Improve Accessibility

 The literature suggests that Thailand fares badly in terms of cancer drug accessibility by global standards. According to PhRMA, among 120 new cancer drugs launched worldwide and approved by the FDA, EMA, and/or Pharmaceuticals and Medical Devices Agency between 2012 and 2021, most debut first in the United States with an average launch lag of zero months, achieving coverage of up to 99% of all newly introduced drugs. Meanwhile, it takes an average of 36 to 39 months for the same set of drugs to enter the Thai market, for both cancer and rare disease medicines alike.^7^ Luksameesate et al showed that, based on 269 cancer drugs (with unspecified screening criteria), 44.2% of cancer drugs (119 out of 269) are not available in Thailand, and 50.7% of accessible cancer drugs (76 out of 150) are not reimbursable.^8^

 Policy-makers in Thailand need to develop effective strategies to enhance patients’ accessibility to innovative cancer drugs, whether on a provisional or regular basis. Even though the Thai government should support innovative medicines that reflect its demographic characteristics—such as disease prevalence and genetic traits—for the sake of accessibility, some countries tend to grant preferential consideration to new drugs that have been approved by the FDA, EMA, and/or Pharmaceuticals and Medical Devices Agency, but have not yet completed the full regulatory process in their own jurisdictions. This approach often takes the form of expedited procedures or conditional approvals, mostly initiated by regulatory bodies in the Middle East and North Africa region.^9,10^

 South Korea, for instance, refers to the pricing levels in eight countries (US, UK, Germany, France, Italy, Switzerland, Japan, and Canada) when making their pricing and reimbursement decisions for new drugs.^11^ Although it is of paramount importance that effective and safe drugs relevant to the Thai population be qualified for financial support through due process, granting conditional approval to drugs already cleared by other regulatory agencies could play a greater role in improving patient accessibility in Thailand.

 Possible alternative approaches include CDFs, managed entry agreements, Risk-Sharing Agreements, exceptional pharmaco-economic evaluation rules—such as flexible incremental cost-effectiveness ratio (ICER) thresholds or pharmaco-economic waivers—and fast-track approval processes.^12^ Evaluation criteria should consider factors such as the life-saving potential of a drug, the severity or rarity of the disease, the existence of alternative treatments, proven clinical benefits, affordability, and availability in other countries.^12^ Moreover, definitions of “life-saving” and “high-cost” drugs should be explicitly stated by national authorities, together with clear procedures for the reappraisal of conditionally approved and CDF-funded drugs.

## Cost-Effectiveness and Managing Cost-Ineffective but Essential Drugs

 An additional challenge lies in integrating cost-ineffective yet clinically indispensable cancer drugs—particularly those with no therapeutic substitutes—into the healthcare system. The discussion must address how Thailand can responsibly handle drugs that are life-saving but would not meet conventional cost-effectiveness thresholds. Global evidence suggests that cost-effectiveness plays a pivotal role in determining access.

 Interestingly, it should be noted that the overall accessibility and the pool of cancer drugs funded by CDFs do not necessarily go hand in hand.^8^ England, for example, has the largest number of cancer drugs managed under the CDF mechanism—35.3% (95 out of 269)—yet 24.9% (67 out of 269) of all cancer drugs remain inaccessible. Meanwhile, Italy provides access to the full list of 269 cancer drugs considered, but only 6.7% (18 out of 269) are reimbursable through its dedicated drugs fund. This apparent paradox largely reflects structural differences in the two reimbursement systems. England operates one of the most stringent health technology assessment frameworks worldwide, meaning that many cancer medicines included in the CDF ultimately fail to satisfy NICE’s cost-effectiveness thresholds, resulting in a lower overall access rate. In contrast, Italy applies more flexible evaluation criteria, allowing most innovative medicines to enter routine reimbursement, which naturally produces a much higher access rate despite the smaller size of its dedicated fund. This structural feature explains why England’s overall access rate appears lower despite its large CDF. Thailand must therefore consider a policy mechanism that balances cost-effectiveness principles with ethical obligations to treat patients with no viable alternatives. Flexibility in ICER thresholds, conditional reimbursement arrangements, or dedicated pathways for ultra-rare or high-severity cases may be required.

## Effectiveness and Operational Structure of Cancer Drug Funds

 Thailand’s policy choices must be understood within the constraints of its socioeconomic structure and the design of its universal health coverage system. The country operates three major public insurance schemes—the Universal Coverage Scheme, Social Security Scheme, and Civil Servant Medical Benefit Scheme—each with distinct financing mechanisms, benefits packages, and reimbursement rules. Fiscal capacity remains limited, with the national drug budget tightly constrained and allowing little flexibility for high-cost or rapidly emerging innovative therapies.^12^ In addition, Thailand’s RWD infrastructure is still fragmented across hospitals and insurance schemes, posing challenges for generating RWD and real-world evidence (RWE) to support managed access arrangements such as CDFs, managed entry agreements, or conditional reimbursement.^13^ These structural factors should guide the design of any dedicated fund for cancer medicines.

 The design and structure of CDFs, including whether RWD are fed back into the regular healthcare system, should be developed so that they promote better health and socioeconomic outcomes. RWD/RWE can serve as complementary, data-driven testing grounds for innovative cancer drugs, while ongoing randomized controlled trials remain the primary reference data source for evaluation. Angelis et al argue that the use of a specialized funding pool should be limited to cases in which clinical uncertainties can be resolved through RWD/RWE within a predefined timeframe.^14^

 Authorities should also determine the extent to which the operational details and resulting data of CDFs are made available to the public. Angelis et al further emphasize that any data collected throughout the process should be publicly accessible for independent analysis.^14^ However, another line of criticism highlights the lack of transparency in the operation of CDFs, including the non-disclosure of ICERs and costs of CDF-funded drugs.^15^

 For the sake of public accountability and patient welfare, self-correcting safeguards should be established by clearly defining coverage periods and interim evaluation mechanisms that allow for the discontinuation of treatments falling below pre-specified thresholds after a set period of time. Consideration should also be given to the idea that CDF budgets be allocated in proportion to the number of cancer patients unable to access existing treatments—or to the unmet segment of the oncology market—to ensure fairness.

## Conclusions

 Thai policy-makers should carefully design a well-structured managed-access fund tailored to the country’s socioeconomic context and aligned with existing institutional and policy frameworks. Discussions on improving cancer drug accessibility should follow a logical progression that includes a clear definition of innovative cancer drugs—particularly in terms of measurable clinical benefits such as OS and cost-effectiveness—consideration of alternative policy options for managing high-priced cancer drugs, integration of cost-ineffective but life-saving drugs with no therapeutic substitutes into the system, and evaluation of the effectiveness of CDFs, supported by empirical evidence, key operational determinants, and a data governance framework. Such a framework could serve as a foundation for Thailand’s future efforts to balance innovation, affordability, and equity in cancer care.

## Disclosure of artificial intelligence (AI) use

 Not applicable.

## Ethical issues

 Not applicable.

## Conflicts of interest

 Author declares that he has no conflicts of interest.

## Disclaimer

 The views expressed in this commentary are solely those of the author and do not necessarily represent the positions of any affiliated institution.
